# Microarray reveals complement components are regulated in the serum-deprived rat retinal ganglion cell line

**Published:** 2007-02-28

**Authors:** Abdelnaby Khalyfa, Timothy Chlon, He Qiang, Neeraj Agarwal, Nigel G.F. Cooper

**Affiliations:** 1 Department of Anatomical Sciences and Neurobiology, University of Louisville, School of Medicine, Louisville, KY; 2 Department of Biochemistry and Molecular Biology, University of Louisville, School of Medicine, Louisville, KY; 3 Department of Cell Biology and Genetics, University of North Texas (UNT) Health Science Center, Fort Worth, TX

## Abstract

**Purpose:**

Glaucoma is a progressive eye disease that leads to blindness due to loss of retinal ganglion cells (RGCs). There are difficulties in using primary cultures of purified RGC to study this pathophysiology. RGC-5, a transformed not RGC line, expresses several markers characteristic of the RGCs. The aim of this study was to generate a genome-wide gene expression of RGC-5 following serum deprivation and to identify candidate genes that may be involved in the signal transduction pathways.

**Methods:**

Apoptosis in the transformed rat RGC-5 was induced by serum deprivation for 0, 8, 24, 48, and 96 h. Briefly, 400 ng of RNA from each sample was reverse transcribed and labeled with Cy3 dye. Fragmented fluorescent cRNA was mixed with hybridization buffer and incubated at 60 °C for 16 h. Labeled cRNA was hybridized to Rat Genome Oligonucleotide Arrays. These arrays contain 22,775 transcripts with one oligonucleotide per transcript (60-mer). Gene expression from scanned images was quantified and analyzed using ArrayVision software. Reproducibility among triplicate arrays was determined by ANOVA statistical analysis. Significant differences in gene expression between apoptotic and nonapoptotic cells were determined based on p-values.

**Results:**

Of the 22,775 transcripts present on the arrays (Agilent rat genome, 60-mer), 713 (8 h), 1,967 (24 h), 1,011 (48 h), and 1,161 (96 h) were differentially expressed relative to the 0 h time point (p-values <0.05). Twenty-three transcripts were common to 8, 24, 48, and 96 h and 130 transcripts were common to the 24, 48, and 96 h time points. The two most highly upregulated genes were *Fdft1* and *Lgals3* (8 h), C3 and *Fcgrt* (24 h), C and *Lcn2* (48 h), and *Mgp* and C3 (96 h). A subset of the differentially expressed genes identified in microarray data (*Ftl1, C3, C1s, Neu1, Polr2g, Acadm, Nupr1, Gch, Dia1, DNase1, Tgfb2*, and *Cyr61*) were validated using quantitative real time polymerase chain reaction (QRT-PCR). Here we show that complement factor H (CFH), the major inhibitor of the alternative complement pathway is downregulated in serum-deprived RGC-5. CFH protein was detected within RGC-5 cells as well as the rat retina with the aid of immunocytochemistry and confocal microscopy.

**Conclusions:**

This study was undertaken to generate a genome-wide gene expression profile of RGC-5 after serum deprivation, and to identify candidate and novel genes that may be involved in the signal transduction pathways leading to apoptosis. RGC-5 serum deprivation revealed up-and downregulation in gene expression profiles. The data gathered from this study was the first report that the genes identified in microarray data and validated by real-time RT-PCR may play an important role in RGC-5 cell death. Among the validated genes, C3 and *C1s* showed significant upregulation of the complement component pathway. The results further indicate that components of the complement pathway are present in neurons of the rat retina. The data indicated that complement factors are likely involved in the pathway leading to ganglion cell death in the serum-deprivation paradigm, which may be similar to the mechanism of cell death in glaucoma.

## Introduction

Glaucoma, the second leading cause of blindness in developed countries [1], is characterized by progressive damage of the optic nerve associated with a selective loss of the retinal ganglion cells [2]. The precise mechanisms involved in glaucoma have yet to be determined, but it is widely accepted that a better appreciation of the factors involved in ganglion cell death is central to the future development of an overall strategy for treatment [3,4].

Animal disease models have long been used as surrogates for human diseases and have been informative. In vivo models with elevated intraocular pressure (IOP) have enabled, apoptosis of retinal ganglion cells to be observed in rats [3] and monkeys [4]. These models are probably good representations of the situation seen in glaucomatous patients [5]. In such models, several studies looked at the mechanisms of pressure-induced optic nerve damage [6], selective loss of ganglion cell function in rats with experimental glaucoma [7], and the anatomy and pathophysiology of the optic nerve head in glaucoma [8]. However, in vivo models may not represent the only approach to study a complex problem in which multiple factors are likely involved.

Several other experimental models have been used to initiate and study ganglion cell death, including direct damage to the rat optic nerve [3,9] and exposure to elevated concentrations of glutamate or its analogues [10,11]. A reduction in the level of neurotrophic factors [2,12] and the possible overexposure to glutamate [13,14] have received recent attention. Both conditions have been shown to affect the survival of retinal ganglion cells (RGCs), and therefore, are implicated in the pathophysiology of RGC cell death in glaucoma. While the relationship of excitotoxicity to glaucoma has been controversial, Ullian et al. [15] recently confirmed the likelihood of a connection between glaucoma and elevated levels of glutamate in the retina [16].

In some cases, isolated as well as purified ganglion cells have been used to explore the pathophysiology of cell death [17,18]. However, the use of primary cells in culture may also be problematic for larger scale studies because of the limited lifespan of the culture, the potential contamination problems [19,20], and the limited yields [21]. To overcome such problems, a permanently transformed RGC line (RGC-5) was recently established [22,23]. RGC-5 cells have been shown to have some [22], but not all [24], of the phenotypic properties of RGCs. We have used the RGC-5 line to look for prospective factors that may be involved in retinal ganglion cell death.

Advances in genomics and microarray technology provide an excellent opportunity to examine global changes in retinal gene expression profiles in diseases and models of diseases [25,26]. DNA microarrays have been utilized in many applications for identifying changes in gene expression patterns including neurodegenerative diseases [27,28]. Microarrays have already been used to investigate human astrocytes cultured from glaucomatous and normal optic nerve heads [29]. They have also been used to construct a custom cDNA microarray of a human retina [30],  to evaluate gene expression patterns in the rat nervous system [31],  and to identify patterns of retinal mRNA expression after experimental elevation of IOP in a rat glaucoma model [32].

In this study, we used 60-mer oligonucleotide-based spotted microarrays, which are known to have good sensitivity under various hybridization conditions [33]. The sensitivity of the 60-mer oligonucleotide microarrays is reported to be about two to eight times [33,34],  better, when compared to the 25-mer arrays, partly due to the larger area available for hybridization. Microarray experiments are now routinely used to collect large-scale data over time which can facilitate the quantitative analysis of genetic regulatory processes [35].

Maher and Hanneken [36] concluded that the RGC-5 line is an excellent model for studying mechanisms of RGC death in response to oxidative stress and for the identification of neuroprotective compounds. We utilized the oligonucleotide-60-mer microarrays to investigate the global pattern of gene expression upon serum-deprived induction of RGC-5 cell death and compared the results to those reported earlier for this model [23]. The results presented in this paper were compared with purified rat retinal ganglion cells (cDNA screening library) [18] and with a recent microarray study of the rat retina in which elevation of IOP was used to evaluate gene expression [32].

## Methods

### Cell culture

Cultures of RGC-5 cells were maintained in growth medium containing low-glucose Dulbecco's modified Eagle's medium (DMEM) containing 10% fetal calf serum (FCS), 100 U/ml penicillin, and 100 μg/ml streptomycin (Sigma-Aldrich, St. Louis, MO) in a humidified atmosphere of 95% air and 5% CO2 at 37 °C, as described by Krishnamoorthy et al. [22,23]. The cells were subcultured every 48 to 72 h with a doubling time of about 18-20 h. To induce apoptosis, the growth medium was withdrawn (serum starvation) and cells were maintained in DMEM for 0, 8, 24, 48, and 96 h. Total RNA was extracted from each time point and maintained at -80 °C until used for analysis. The source of RGC-5 cells was provided by Dr. Agarwal.

### Preparation of DNA fragmentation

Apoptotic cells typically produce a ladder of nucleotide fragments that can be easily visualized by agarose gel electrophoresis. DNA fragmentation was isolated and carried out as previously described by Fujikawa et al [37]. We adapted these procedures by extracting genomic DNA from apoptotic and nonapoptotic RGC-5 samples by collecting both floating and attached cells using centrifugation. These cells were harvested and rinsed with ice-cold phosphate buffered saline (PBS). The pellets were solubilized in 390 μl lysis buffer (30 μl of 10 mM Tris-HCl, pH 8.0); 60 μl of 100 mM sodium chloride, 150 μl of 25 mM EDTA; (150 μl of 0.5% SDS) and 0.4 mg/ml proteinase K. They were then incubated at 55 °C overnight in a rotor shaker. Samples were extracted twice with phenol: chloroform (1:1) and once with chloroform. DNA was precipitated using one volume of 3 M sodium acetate, pH 5.2 and cold 100% ethanol, followed by washing with 70% ethanol. Pellets were dried using a refrigerated Speed Vac concentrator (Savant, Pittsboro, NC) and dissolved in 1X TE buffer. DNase-free RNase was added to the samples to a final concentration of 100 μg/ml and incubated at 37 °C for 3 h. The samples were incubated for 30 min at 37 °C followed by 5 min at 65 °C. DNA was subjected to electrophoresis in 2% agarose gel, and the bands were visualized with the aid of ultraviolet (UV) irradiation after ethidium bromide staining.

### RNA extraction

Total RNA was extracted from the RGC-5 cells using spin columns (RNeasy; Qiagen, Valencia, CA), followed by DNase treatment according to the manufacturer's instructions. The quantity and purity of total RNA for samples were analyzed with spectrophotometry readings at 260/280 nm. The integrity of intact total RNA was verified with an Agilent 2100 Bioanalyzer (Agilent Technologies, Palo Alto, CA), and the RNA Integrity Numbers (RINs) for the samples were obtained. For routine RNA quality control analysis, Agilent's 2100 bioanalyzer with an RNA 6000 Nano LabChip® Kit (Agilent) was used. RNA samples were each prepared to a concentration of 25 ng/μl in parallel to a 6000 RNA ladder (Ambion, Houston, TX). The range of 28S/18S ribosomal RNA was typically 1.8-2.1.

### cRNA preparation

A total of 15 samples were used for cDNA synthesis, three separate biological samples of total RNA from RGC-5 for five time points. Equal quantities of total RNA from the samples were labeled using Agilent low RNA input fluorescent linear amplification kit, and hybridized to three independent identical arrays of Agilent oligonucleotide probe sequences. Briefly, total RNA (400 ng) was reverse transcribed into cDNA, using Moloney murine leukemia virus (MMLV) reverse transcriptase with oligo(dT) promoter primer, and incubated at 40 °C for 2 h. Fluorescent cRNAs were synthesized by in vitro transcription using T7 RNA polymerase and labeled with cyanine 3-labeled dCTP (PerkinElmer, Boston MA). Labeled cRNAs were further purified using RNase mini purification columns (Qiagen) to remove unlabeled products. Fifteen picomoles of the fluorescently labeled cRNAs were used for each of the microarray hybridizations.

### Microarray hybridization

The hybridization solution was prepared using the in situ hybridization kit plus (Agilent). The Agilent 60-mer array (G4130B) contains 22,775 rat probe sequences which include 162 negative controls and 913 positive controls. Each array contains one probe sequence (60-mer) per transcript spotted using a noncontact spotter onto specially prepared glass slides. Hybridizations were performed using the manufacturer's oligo processing protocols (Agilent). About 0.75 μg of labeled cRNA and 5 μl of 25X fragmentation buffer were incubated at 60 °C for 30 min. Hybridization was carried out in 490 μl of a hybridization mixture at 60 ° for 17 h in Agilent's microarray hybridization chambers according to the Agilent protocol. The 15 microarrays slides representing 15 samples were washed for approximately 30 s each in 6x SSPE, 0.005% sarcosine and 0.006x SSPE.

### Digitization of images and statistical analysis

The arrays were scanned immediately after hybridization with a pixel resolution of 10 μm using a laser Typhoon 9410 scanner (GE HealthCare, Piscataway, NJ) that excites the Cy3 (green) fluorophore at optimal wavelengths of 532. Arrays were scanned at different photomultiplier tube (PMT) voltages (500-700 PMT). PMT setting of 600 nm was found to be optimal for signal detection analysis based on negative and positive controls that were included in the array platforms. Images were captured and saved by ImageQuant software v. 5.2 (GE HeathCare, Piscataway, NJ). Images were then loaded into ArrayVision v.8.0 software (Imaging Research Inc., Ontario, Canada) to calculate the median intensities for each spot of Cy3 fluorescent signals.

The net intensity of a spot (one gene) was calculated by subtracting the median of the background intensities from the median of the chip replicates spot intensities. These background-corrected intensities were then normalized by the median normalization method. The fold change values for the differentially expressed genes were calculated from ratios of intensities between the two time points designated as test groups. The t-test p-values (in which two-sample unequal variances were used) were utilized to detect the significance of differences between the two test groups. Log ratios of intensities for individual genes were determined for each test/control (serum-deprived/nondeprived) group, from which the mean value of log ratios for each sample group was also obtained.

### Biological pathways analysis

Differentially expressed genes, which had RefSeq annotations were subsequently analyzed using PathwayAssist software (Iobion Informatics, LLC, La Jolla, CA). We constructed tables showing gene associations with cellular processes, as well as figures showing the association of altered genes with more complete gene-regulatory networks.

### Real-time polymerase chain reaction

Quantitative Real-Time PCR values (Applied Biosystems; 7300 instrument) of the differentially expressed genes detected in serum-deprived (8, 24, 48, and 96 h) and nondeprived (zero time point) RGC-5 cells were used to validate the microarray results. The primer pairs for each cDNA were obtained from Applied Biosystems demand-assay. Briefly, the sequences for each selected gene from microarray results were used to conduct a BLASTN search against *Rattus norvegicus* (rat) on the NCBI database. The sequences of primers designed were selected to be within the same region of the gene used to develop the microarray sequence probes. Two micrograms of total RNA from deprived and nondeprived samples were used to generate cDNA templates for RT-PCR. cDNA synthesis was performed using a High-Capacity cDNA Archive Kit (Applied Biosystems). The first strand cDNA products were further diluted 20-50 fold and used as PCR templates. The TaqMan® Master Mix Reagent Kit (Applied Biosystems) was used to amplify and quantify each transcript of interest in 40 μl reactions. To ensure specific amplification, various negative controls in the PCR reaction. Triplicate PCR reactions were performed in 96-well plates for each gene in parallel with the 18S rRNA. The steps involved in the reaction program included in the following: the initial step of 2 min at 50 °C; denaturation at 95 °C for 10 min, followed by 40 thermal cycles of denaturation (15 s at 95 °C), and elongation (1 min at 60 °C). Quantitative values are obtained from the cycle number (Ct value) using the Biosystems analysis software. The threshold cycle (C_T_) values were averaged from the values obtained from each reaction, and each gene was normalized to 18S rRNA level. Each gene of interest and 18S rRNA were tested in triplicates to determine the Ct-difference. These Ct values were averaged and the difference between the 18S Ct (Avg) and the gene of interest Ct (Avg) was calculated (Ct-diff). The fold change was calculated by the 2-Δ^CT^, where ΔΔCT=(C_T_apoptotic-C_T_non-apoptotic). C_T_ apoptotic means the signal of an individual gene expressed in time course samples (8, 24, 48, and 96 h), while C_T_non-apoptotic means the signal of the same gene expressed in 0 h. The relative expression of the gene of interest was analyzed using the 2-ΔΔ^CT^ method [38].

### Confocal microscopy

Whole eyes of adult Sprague-Dawley rats were isolated, fixed in 4% paraformaldehyde, embedded in paraffin, sectioned at a thickness of 5 μm, and deparaffinized prior to immunocytochemical treatment. RGC-5 cells were fixed in 4% paraformaldehyde and rinsed in PBS; pH 7.4. The sections or cells were then immersed in a 0.1 Triton X-100 solution for 30 min and rinsed in PBS at least three times for 5 min each. Samples were then treated with a 0.05 M solution of glycine for 15 min to eliminate free aldehydes. The sections and cells were subsequently incubated in a blocking solution of 5% bovine serumm albumin (BSA), and 10% normal serum of the host animal for the secondary antibody was also in PBS for 15 min. This was followed by an overnight incubation at 4 °C with primary antibodies for factor H (Santa Cruz Biotechnology, Santa Cruz, CA) diluted to 1 μg/ml in a solution of 1% BSA and 1% normal serum of secondary antibody host animal in PBS. For controls, the primary antibody was omitted and replaced by goat IgG at the same concentration as the primary antibody. After incubation, the study samples were rinsed four times in PBS and incubated with the secondary, antibody (goat) coupled to Alexa green 488 (Molecular Probes, Inc) diluted 1:200 in buffer for 1 h at room temperature. The sections and cells were then rinsed in PBS. To visualize the nuclei, we stained the sections and cells with Hoechst nuclear stain (Hoechst number 33342 trihydrochloride trihydrate, Invitrogen) diluted according to the manufacturer's recommendation for 5-10 min at room temperature in the dark. The sections and cells were again rinsed in PBS and double distilled water, mounted with antifade mounting media, and examined with a Zeiss LSM410 confocal microscope (Carl Zeiss Microimaging, Inc., Thornwood, NY) with a 40X water immersion objective (numerical aperture 1.2). Samples were excited at 488 nm (for Alexa 488) using an argon ion laser (Melles Girot/Omnichrome, Carlsbad, CA) and fluorescence collected with a 510-525 nm emission filter. Hoechst nuclear stain was excited at 364 nm with a UV laser (Coherent Innova Technology, Santa Clara, CA), and fluorescence emission was collected using a 400-440 nm emission filter.

## Results

### Microarray gene expression

**Figure 1:**
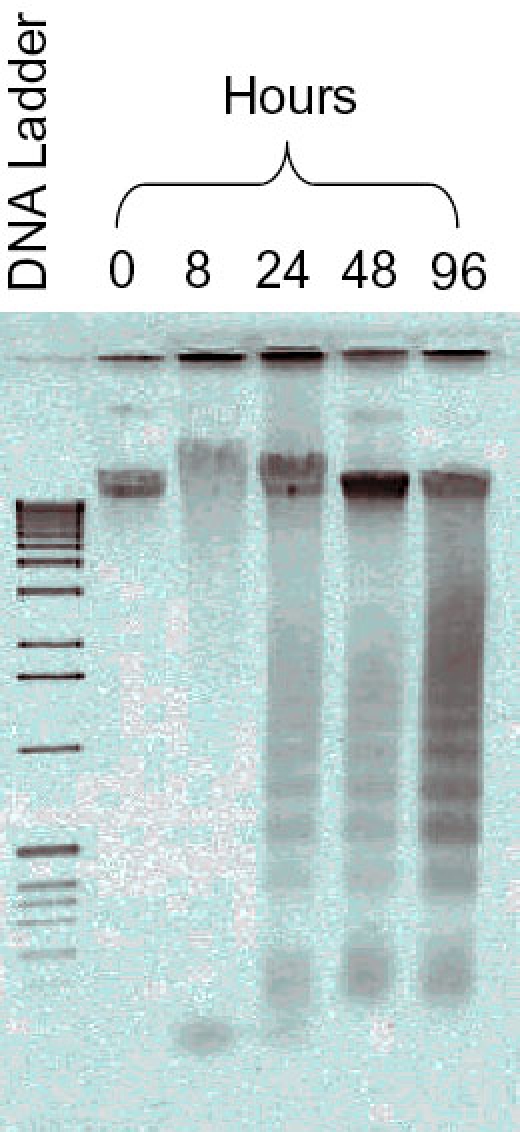
Apoptotic DNA fragmentation in serum-deprived retinal ganglion cell-5 samples. DNA was extracted and electrophoresed in parallel on the same agarose gel with ethidium bromide staining. Lane 2 (0 h), and lane 3 (8 h) show a lack of DNA fragmentation, but lanes 4, 5, and 6 show a ladder of internucleosomal DNA fragments. In dying cells, DNA is cleaved by an endonuclease that fragments the chromatin into nucleosomal units, which are multiples of about 180-bp oligomer at about 200 bp. Molecular weight standards are shown on lane one on the same gel.

Comprehensive gene expression profiles were examined to identify genes associated with apoptosis and signaling transduction pathways in serum-deprived RGC-5 cells. DNA fragmentation was used to confirm that the serum-deprived RGC-5 cells (0, 8, 24, 48, and 96 h) were dying. The results showed that the level of DNA laddering gradually increased with time in the serum-deprived condition (Figure 1). The data presented showed fragmentation of DNA into different length pieces (180 bp). In addition, no DNA fragmentation was observed in the control culture (0 h) compared with the other serum-deprived cultures, thereby confirming that this sample can serve as a control sample. These results indicated that the number of the dying cells in the serum-deprived condition increased with time. It was noted that there were scant levels of DNA fragmentation in the 8 h samples. The graded accumulation of cell death in the serum-deprived RGC-5 cells allowed us to analyze changes in gene expression levels at these different time points.

**Figure 2:**
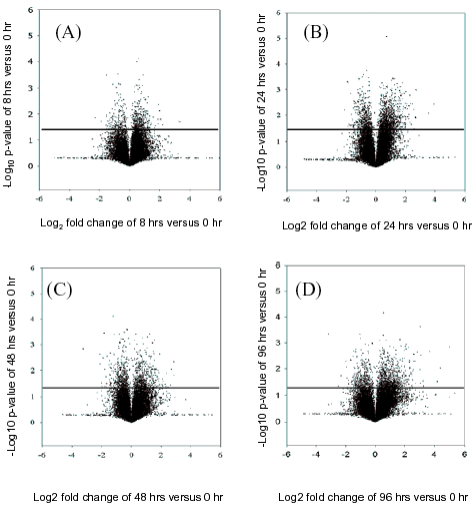
Volcano plot of normalized median intensities of retinal ganglion cells serum-deprived. The Volcano plots show differentially expressed genes at 8 h versus 0 h (**A**), 24 h versus 0 h (**B**), 48 h versus 0 h (**C**), and the 96 h versus 0 h (**D**) samples. The gene-specific F-test (y-axis) denoting significance for each gene is represented as individual spot (Σ). The Volcano plots allowed the visualization of fold-change and statistically significant p-values at the same time. Thus, the statistical significance of either large or small fold changes can be seen. Volcano Plots show genes that have significantly different expression between two sample types based on both biological difference (absolute log2 (estimated fold change) greater than some constant threshold) and statistical difference (-log10 (p value) greater than some constant threshold). Volcano plots have a "V" shape appearance so there will usually be no genes with small biological effect (i.e., log (fold change) close to 0) with high statistical significance.

Volcano plots were used to determine the most significant altered expression of genes based on both traditional fold changes and statistical p-values (Figure 2). This figure shows the log_2_ fold change of normalized intensity versus their log_10_ p-values for 4 time points relative to the zero time point. These data include 8 h versus 0 h (Figure 2A), 24 h versus 0 h (Figure 2B), 48 h versus 0 h (Figure 2C), and 96 h versus 0 h (Figure 2D). Spots in the extreme upper left and right corners of the volcano plots marked the largest, and most statistically significant, changes in gene expression. For each gene present, the volcano plot consisted of a plot containing log_2_ of the fold change for two conditions on the *x* axis and the negative log10 of the p value on the *y* axis. The dots above the horizontal line indicated the p-value of <0.05 for gene expression for both annotated and nonannotated genes. For example, in 8 h versus 0 h samples, the log_2_ at a scale of 1 showed a 2 fold change on the X axis, and -log_10_ at a scale of 1.3 on the Y axis equal to a p-value of 0.05. Therefore, based on the fold change and p value the horizontal line indicates the changes in gene expression values used in this study. This procedure was used to identify changes in gene expression at all time points.

### Identification of differentially expressed genes

Of 22K transcripts, 713 transcripts (8 h); 1,967 transcripts (24 h); 1011 transcripts (48 h), and 1,162 transcripts (96 h) were found to be differentially expressed relative to the control (0 h) condition. From the differentially expressed data (Figure 2), we prepared a list of the annotated (RefSeq accession numbers) and nonannotated (EST accession numbers) transcripts. For the annotated genes, there were 169 genes for 8 h/0 h, 418 genes for 24 h/0 h, 217 genes for 48 h/0 h, and 153 genes for 96 h/0 h. Based on the p-value cutoff (<0.05), a list of 23 differentially expressed transcripts whose expression was either up- or downregulated were identified as common to all 4 time points (8, 24, 48, and 96 h) as indicated in Table 1. Of these 23 transcripts (Table 1) there were 7 transcripts with RefSeq accession numbers (annotated) and 16 ESTs (nonannotated). In this table there were a number of genes that were consistently upregulated including DNnase I (NM_013097), rat cDNA clone (AI071183), and normalized rat brain, transcribed locus (AI229209). Of the 23 genes there were 10 genes that were upregulated using the stringent criterion (p<0.05) for significance. This stringency was used to minimize the prospect of obtaining false positives.

**Table 1:** Differentially expressed genes in serum deprived at 8, 24, 48, and 96 h.

Fold Change					p-values				Accession Numbers	Description
0hr/0hr	8hrs/0hr	24hrs/0hr	48hrs/0hr	96hrs/0hr	8hrs/0hr	24hrs/0hr	48hrs/0hr	96hrs/0hr		
1	0.34	0.41	0.28	0.34	0.000	0.020	0.000	0.037	NM_031327	cysteine rich protein 61 (Cyr61)
1	0.71	0.61	0.72	0.52	O.OO8	O.OOI	0.009	0.049	CB545090	similar to ATP synthase, subunit b-like
1	0.40	0.41	0.54	0.63	0.009	0.006	0.014	0.044	AW528454	Simi lar to hypothetical protein FLJ12484
1	2.31	3.15	3.01	4.19	0.010	0.013	0.012	0.012	BQ7 82630	Similar to apoptosis related protein APR-3;
1	0.54	0.36	0.23	0.38	0.013	0.033	0.006	0.010	AA892839	similar to Mouse, similar to signal peptidase
1	0.21	0.35	0.23	0.35	0.014	0.005	0.002	0.003	A1029745	Transcribed locus
1	4.15	6.57	5.09	5.99	0.016	O.OOI	0.013	0.011	BQ201826	similar to Mouse eye! in G2 (Ccng2) gene.
1	0.40	0.77	0.61	0.52	0.017	0.039	0.016	0.048	NM_017023	Potassium inwardly-rectifying channel
1	0.50	0.63	0.71	0.50	0.019	0.031	0.032	0.011	BM387283	unknown function
1	1.71	2.71	1.88	1.79	0.022	O.OOI	0.033	0.031	AW253722	RAB13. member RAS oncogene family
1	1.26	1.46	2.34	18.92	0.027	0.023	0.006	0.028	NM_013097	deoxyri bo nuclease 1 (Dnasel)
1	1.44	1.60	1.25	1.62	0.028	0.011	0.041	0.007	CBS44857	Glucosidase, alpha; acid (Pompe disease.
1	0.72	0.54	0.68	0.64	0.028	0.006	0.044	0.001	CBS46229	Similar to retinitis pigmentosa GTPase
1	2.31	3.42	2.19	3.12	0.029	0.002	0.042	0.012	NM_012915	ATPase inhibitor (rat mitochondrial 1F1
1	0.73	0.65	0.56	0.60	0.033	0.016	0.017	0.024	NM_139336	UDP-glucuronate decarboxylase 1 (Uxsl)
1	2.53	2.72	1.80	2.13	0.033	0.019	0.041	0.043	NM_016986	acyl-Coenzyme A dehydrogenase. C-4 to C-
1	0.35	0.26	0.38	0.20	0.037	0.000	0.014	0.016	BF2S3172	similar to Mouse intronless MDB0109 gene.
1	1.39	1.52	1.79	2.16	0.040	O.OOS	0.047	0.046	AI071183	rat cDNA clone, partial cds
1	1.73	0.72	0.76	0.60	0.042	0.000	0.047	0.006	B1286045	Plakophilin 3 (predicted)
1	2.04	2.11	2.88	3.95	0.045	0.005	0.009	0.019	A1229209	normalized rat brain. Transcribed locus
1	1.87	2.72	1.86	3.19	0.048	0.005	0.031	0.029	BQ205672	similar to Translation (AF289554_1) Homo
1	0.45	0.56	0.36	0.39	0.049	0.020	0.014	0.016	NM_031131	transforming growth factor, beta 2 (Tgfb2)
1	0.49	0.48	0.45	0.44	0.050	0.046	0.030	0.022	BF228368.I	SS'Jr Rattus norvegicuscDNA, mRNA

List of differentially expressed genes whose expression values changes in serum-deprived RGC-5 cells at 8, 24, 48, and 96 h.

The data was reexamined without the 8 h time point to determine if there were significant changes subsequent to this time point. Clearly at the 8 h time point there was scant DNA fragmentation (Figure 1), and consequently, a limited number of differentially expressed genes were observed at this time point. This manipulation led to the identification of 130 transcripts (39 RefSeq and 91 ESTs transcripts) that were commonly detected in the 24, 48, and 96 h time points (Table 2). The data indicated that a large number of known and unknown genes could be identified. Those genes, which have RefSeq accession numbers, were the primary focus for this study because they represented a database of genes that have been documented at a higher level of certainty than those found in other experimental gene collections such as UniGene. The RefSeq annotated genes were used for further analysis and validation.

**Table 2:** Differentially expressed genes in serum deprived at 24, 48, and 96 h.

Ratio				p-value			RefSeq	Symbol	Description
0hr/0hr	24hrs/0hr	48hrs/0hr	96hrs/0hr	24hrs/0hr	48hrs/0hr	96hrs/0hr			
1	0.27	0.11	0.11	0.0005	0.0015	0.0004	NM_053356	Col1a2	procollagen, type I, alpha 2
1	0.35	0.43	0.44	0.0255	0.0351	0.0246	NM_022229	Hspel	heat shock protein 1 (chaperonin)
1	0.36	0.40	0.41	0.0423	0.0465	0.0378	NM_032070	Hmgic	non-histone chromosomal architectural protein HMGI-C
1	039	0.44	0.44	0.0032	0.0229	0.0248	NM_030873	Pfn2	profilin II
1	0.41	0.28	0.34	0.0198	0.0004	0.0368	NM.031327	Cyr61	cysteine rich protein 61
1	0.43	0.36	0.54	0.0344	0.0356	0.0340	NM_019187	Coq3	coenzyme Q (ubiquinone)
1	0.56	0.36	0.39	0.0197	0.0138	0.0159	NM_031131	Tgfb2	transforming growth factor, beta 2
1	0.59	0.40	0.54	0.0198	0.0084	0.0391	NM_019232	Sgk	serunVglucocorticoid regulated kinase
1	0.65	0.56	0.60	0.0161	0.0169	0.0239	NM_139336	Uxsl	UDP-glucuronate decarboxylase 1
1	0.66	0.67	0.45	0.0172	0.0402	0.0417	NM_031603	Ywhae	tyrosine 3-monooxygenase'tryptophan 5-monooxygenase
1	0.69	0.56	0.65	0.0211	0.0267	0.0351	NM_017312	Bok	Bcl-2-related ovarian killer protein
1	0.74	0.55	0.78	0.0020	0.0054	0.0352	NM_012779	Aqp5	aquaporin5
1	0.75	0.58	0.50	0.0011	0.0064	0.0007	NM_ 139060	Csnkld	casein kinase I.delta
1	0.76	0.71	0.67	0.0033	0.0060	0.0218	NM_031014	Alk	5-aminoimidazole-4-carboxamide ribonucleotide formyltransferasc
1	0.77	0.61	0.52	0.0386	0.0162	0.0478	NM.017023	Kcnjl	Potassium inwardly-rectifying channel, subfamily J. member 1
1	1.33	1.49	1.52	0.0230	0.0450	0.0026	NM.012542	Cyp2a3a	Cytochrome P450. subfamily IIA (phenobarbital-inducble)
1	1.46	2.34	18.92	0.0225	0.0055	0.0282	NM_013097	Dnasel	Rattus norvegjeus Deoxyribonuclca.se 1
1	1.59	1.65	3.92	0.0457	0.0178	0.0020	NM_054008	Rgc32	Rgc32 protein.
1	1.7)	2.55	3.86	0.0222	0.0079	0.0269	NM_024356	Gch	GTPcyclohydrolase I
1	1.82	1.44	1.43	0.0039	0.0234	0.0159	NM_013175	Sgnel	Secretory granule neuroendocrine, protein 1 (7B2 protein)
1	1.84	1.93	2.1 S	0.0393	0.0309	0.0280	NM_138877	Dial	Diaphorase(NADH) (cytochrome b-5 reductase)
1	1.89	1.15	1.67	0.0348	0.0874	0.1457	NM_012828	Cacnb3	Calcium channel subunit beta 3
1	1.95	1.42	1.63	0.0353	0.0424	0.0207	NM_O225O0	Fill	ferritin light chain I
1	1.95	2.28	2.22	0.0208	0.0351	0.0315	NM_012578	HlfO	HistoneHl-0
1	2.01	4.26	18.16	0.0087	0.0202	0.0287	NM_031116	Ccl5	chemokine(C-C motif) ligand 5
1	2.12	1.87	2.57	0.0011	0.0384	0.0292	NM_031830	Flot2	flotillin 2
1	2.14	2.75	2.57	0.0320	0.0239	0.0270	NM_053948	Polr2g	polymerase (RNA) 11 (DNA directed)polypeptide G
1	2.23	2.86	9.60	0.0004	0.0023	0.0431	NM_138900	Cls	complement component l.s subcomponent
1	2.26	2.09	2.43	0.0200	0.0306	0.0484	NM_0S0888	Bnip3l	BCL2Adenovirus E IB 19 kDa-interacting protein 3-like
1	2.29	1.84	1.78	0.0389	0.0310	0.0099	NM.012960	Ggh	gamma-glutamyl hydrolase
1	2.55	2.44	2.56	0.0485	0.0032	0.0384	NM.053611	Nuprl	nuclear proten 1
1	2.72	1.80	2.13	0.0186	0.0410	0.0426	NM_016986	Acadm	Acyl-Coenzyme A dehydrogenase, C-4 to C-12 straight-chain
1	2.85	2.32	3.39	0.0197	0.0493	0.0274	NM_017188	Uncll9	UNC-119 homolog (C. elegans)

List of differentially expressed genes whose expression values changes in serum-deprived RGC-5 cells at 24, 48, and 96 h.

### Identification of cell death-related genes

To identify genes that are associated with cell death in the RGC-5 cells, we searched accession numbers in the NCBI database. The number of differentially expressed genes previously reported to be involved in neuronal cell death varied among the time courses as follows: 14 genes (8 h), 19 genes (24 h), 13 genes (48 h), and 23 genes (96 h). Several genes found to be relevant to neuronal cell death were differentially expressed in the RGC-5 cells. These included: mitogen activated protein kinase 8 interacting protein (MAPK8IP) 1.9- fold and death-associated protein 1 (DAP-1, RAP7A), 1.9 fold at 8 h; programmed cell death 4 (PDCD4), 2.3 fold and MAPK8IP (2.8- fold) at 24 h; GTP cyclohydrolase 1 (Gch) 2.6 fold and growth arrest and DNA-damage-inducible 45 (Gadd45) 3 fold at 48 h; Gadd45 (4.7 fold) and deoxyribonuclease (DNase-1; 18.9 fold) at 96 h. After exclusion of the 8 h time point, the data indicated other differentially expressed genes common to the 24, 48, and 96 h time points and included the 5 known neuronal cell death genes: Cyr61, TGFβ, Bok, Bnip3l, and DNase-1. Together, these results indicated that the initiation of apoptosis pathways in RGC-5 cells involved a number of known cell death genes.

### Comparative data analyses

Before comparing the patterns of expression in RGC-5 cells with data from other published databases, we compared our results with a rat retina cDNA EST screening library [18]. In the latter study, the identified clones were obtained from the EST library and linked to human optic neuropathies. We found 44 of the 72 genes identified by Farkas et al. [18] to be specific to the rat (*Rattus norvegicus*). Of the other 28 genes, 26 were specific to the mouse (*Mus musculus*), and two were specifically human (*Homo sapiens*). Twenty-two of the 44 rat genes had altered expression values in our microarray analysis (Table 3). Of the 22 genes that were not present, we determined that most of those genes had accession numbers that conflicted with the accession numbers for genes present in the Agilent microarray.

**Table 3:** Genes identified in our microarray data and other.

	RefSeq	Symbol	Description
1	NM_031100	Rpll0	ribosomal protein L10
2	NM_021261	Tmsbl0	thymosin, beta 10
3	NM_03I099	Rpl5	ribosomal protein L5
4	NM_024351	HspaS	heat shock protein 8
5	NM_053330	Rpl21	ribosomal protein L21
6	NM_012497	Aldoc	C, fructose-biphosphale
7	NM_012963	Hmgbl	high mobility group box 1
8	NM_030873	Pfti2	profilin 2
9	NM_013067	Rpn 1	ribophorin I
10	NM_013216	Rheb	Ras homolog enriched in brain
11	NM_021697	Kcnvl	potassium channel, subfamily V, member 1
12	NM_013226	Rpl32	ribosomal protein L32
13	NM_I73102	Tubb5	tubulin, beta 5
14	NM_017138	Lamrl	laminin receptor 1 (67kD, ribosomal protein SA)
15	NM_031683	Smell I	structural maintenance of chromosomes 1 like I
16	NM_139254	Tubb3	tubulin, beta 3
17	NM_022934	Dnaja 1	DnaJ-like protein
18	NM_03I119	Ssb	Sjogren syndrome antigen B
19	NM_022298	Tubal	tubulin, alpha 1
20	NM_021766	Pgrmc 1	progesterone receptor membrane component 1
21	NM_012570	Gludl	glutamate dehydrogenase I
22	NM_053297	Pkm2	pyruvate kinase, muscle.

Comparison between genes identified in our microarray data and another study reported by Farkas et al. (2004).

We also compared the RGC-5 dataset with a study [32] in which a microarray analysis of the rat retina was used to determine the pattern of gene expression related to elevated IOP. The data derived from examination of the 96 h time point for serum-deprived RGC-5 cells were compared with the published data derived from rat eyes after 35 days of elevated IOP (Table 4). Of the 29 genes with RefSeq accession numbers considered by both studies, 14 genes (*Lcn2, Cd74, C3, Lgals3, C1s, Fcgr3, Arpc1b, Mmp3, Bzrp, Cebpd, Mgp, Cp, Ptpn1*, and *Cntf*) showed similar changes in gene expression. This comparison served to illustrate that a number of altered genes were found in common, although there also appeared to be novel genes identified in each of the experimental paradigms. The latter fact is not surprising because the in vivo studies of the retina represent changes that could have occurred in one or more of the multiple cell types.

**Table 4:** Genes identified in our microarray and compared with other data.

Gene Bank	Accession #	Late Changes, 35 days	Ratio 96hrs/0hr	Description
AA946503	NM 130741.1	25.9	32.1	*Lcn2, lipocalin 2
XI3044	NM 013069.1	19.6	2 0	*Cd74 antigen
X52477	NM 016994.1	10	31.6	*C3, complement component 3
J02962	NM 031832.1	7.9	3.0	*Lgals3, IgE binding prolein
D88250	NM 138900.1	4.7	9.6	*C 1 s, complement component I, s subcomponent
M32062	NM 053843.2	4.3	4.8	*Fcgr3, Fc receptor, IgG, low affinity III
AF083269	NM 019289.2	2.5	2.0	*Arpclb, actin related protein 2/3 complex
X02601	NM 133523.1	3.5	1.9	*Mmp3, matrix metalloproteinase 3
D90404	NM 017097.1	3.4	1.3	Ctsc, cathepsin C
J05I22	NM 012515.1	3.3	1.8	*Bzrp, benzodiazepin receptor
M65I49	NM 013154.1	8.1	7.3	*Cebpd, CCAAT/enhancer binding, protein (C EBP) delta
AA945867	NM 021835.2	4	1.2	c-jun
AF023087	NM 012551.1	3.8	1.4	Egrl, early growth response 1
L23148	NM 012797.1	3.8	1.2	Idl, inhibitor of DNA binding 1
AI0I2030	NM 012862.1	2.6	66.4	*Mgp, matrix C51a protein
AFO17437	NM 019195.2	2.7	1.4	Cd47, integrin-associated protein
XI6072	NM 012937.1		0.7	Crybb2, crystallin, beta B2
AA817854	NM 012532.1	5.9	1.5	*Cp, ceruloplasmin
All 13289	NM 012637.1	3.1	1.5	* Protein-tyrosine phosphatase (Ptpnl)
M96601	NM 017206.1	-2.1	0 8	Slc6a6, solute carrier family 6, member 6
M3I837	NM 012588.1	S	1.0	Igfbp3, insulin-like growth factor binding protein 3
L209I3	NM 031836.1	4.9	1.1	Vegf, vascular endothelial growth factor form 3
AA892559	NM 013166.1	2	1 6	*Cntf, ciliary neurotropic factor

List of differentially expressed genes obtained from our microarray compared with other data reported by Ahmed et al. (2004). The genes show similar fold changes between our study and other study are labeled by asterisk [32]. The selected genes were chosen to be 1.5-fold change or more.

### Identification of signaling pathways

**Figure 3:**
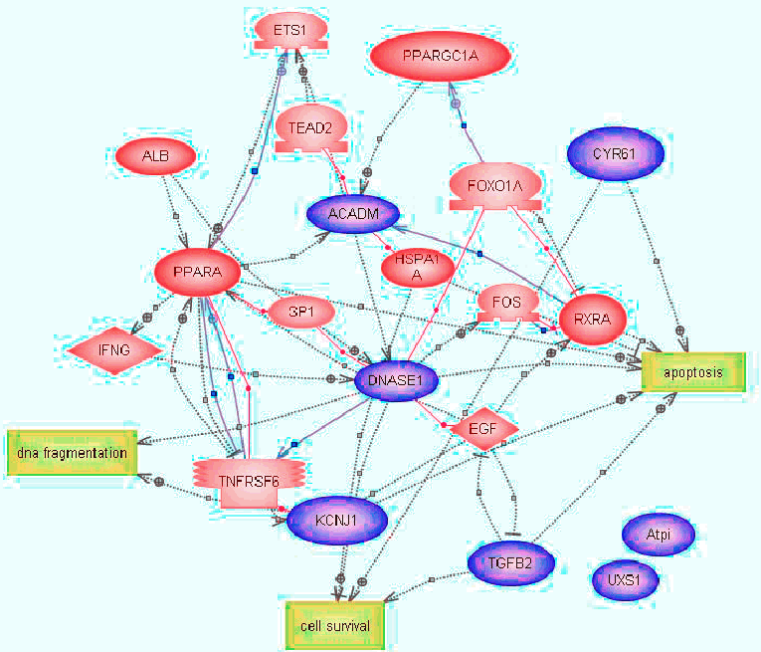
Schematic representations of potential signaling pathways involved in serum-deprived retinal ganglion cell-5 cells. Pathways were identified by incorporating the microarray results (differentially expressed genes identified at 8, 24, 48, and 96 h of serum deprivation) into PathwayAssist software. The pathway connects the proteins (blue color) and cell processes together. Three major biological processes of cell survival, apoptosis, and DNA fragmentation, regulated by these genes are represented by yellow color squares.

To identify potential signaling and pathways and gene-regulatory networks that are associated with retinal ganglion cell death,we imported the microarray expression data to PathwayAssist (Iobion, Informatics, LLC). Biological pathways were identified using genes differentially expressed at: (1) 8, 24, 48, and 96 h, and (2) 24, 48, and 96 h serum deprivation. Only the genes containing RefSeq accession numbers were used for this study because these genes are well annotated and less redundant compared to other databases. The data (Figure 3) showed the signaling pathways for the genes that were differentially expressed and common to the 8, 24, 48, and 96 h time points. Of the seven annotated genes, only five of them (Cyr61, TGFβ, Bok, Bnip3l, and DNase 1) were identified in 8, 24, 48, and 96 h. The objective of this experiment was to find other candidate genes in gene-regulatory networks. We found 13 candidate genes associated with the five genes identified by the microarray analysis using PathwayAssist. The total of identified 18 genes led to the further identification of three cellular processes which included cell survival, apoptosis, and DNA fragmentation. Two genes (*Uxs1*, and *ATPIF1*) were found not to be connected to any other genes in this pathway.

**Figure 4:**
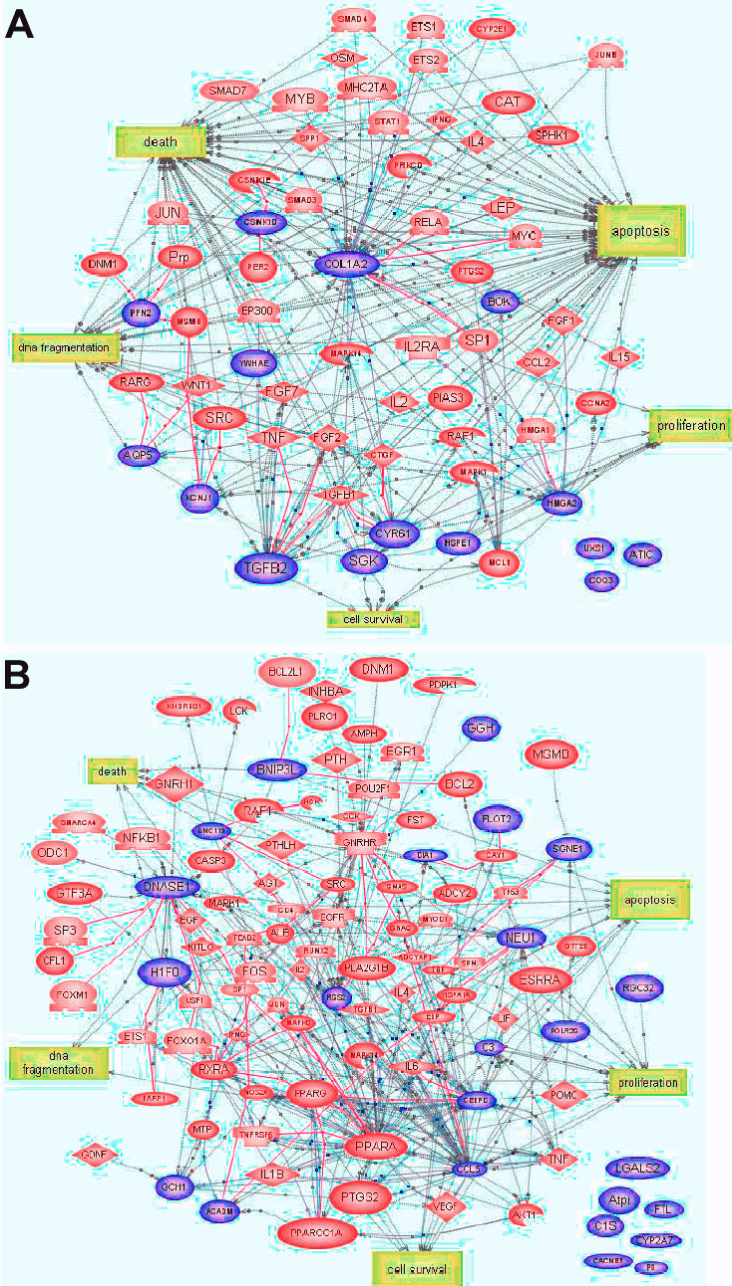
The biological pathway of the differentially expressed genes at 24, 48, and 96 h serum deprivation. **A**: Genes downregulated by serum deprivation (blue color). **B**: Genes upregulated by serum deprivation (violet color). The pathway was constructed on PathwayAssist software by searching for the shortest path to connect the genes of interest by other genes or cell processes with which they interacted through expression or regulation only. Each of these additional nodes has documented relation to apoptosis. The genes highlighted in blue color are the genes identified by microarray analysis, and the genes in red ovals diamonds color are the potential target genes identified with the aid of PathwayAssist.

After removal of the 8 h time point as described, 15 significantly down-regulated genes were found to be common to the 24, 48, and 96 h time points, and these were used to build a second signaling pathway (Figure 4A). Twelve (*Col1a2, Hsp60, Pfn2, Hmgic, Cyr61, Tgfb2, Sgk,Ywhae, Bok, Cqp5, Csnk1d, Kcnj1*) of these could be used to construct a pathway, which included 49 additional target genes. A total of 61 genes were found to be associated with five cellular processes which included DNA fragmentation, apoptosis, cell death, proliferation, and cell survival.

In addition, there were 24 significantly upregulated genes at the 24, 48, and 96 h time points, and there were four cell processes identified in PathwayAssist (apoptosis, cell death, cell survival, proliferation, and differentiation). We were able to use 17 (*Cyp2a3a, Sgne1, Dnase1, Rgc32, Gch, Sgne1, Dia1, H1f0, Scya5, Flot2, Polr2g, Bnip3l, Ggh, Acadm, Uncl19, Cebpd, Rgs2, Neu1, C3*) of these 24 genes to construct a pathway with an associated 81 additional target genes (Figure 4B). These 98 total genes were related to cellular processes, which included DNA fragmentation, apoptosis, cell survival, and proliferation. Thus, we have identified a series of interacting genes that constitute a potentially important gene-regulatory network related to cell death and survival pathways.

**Figure 5:**
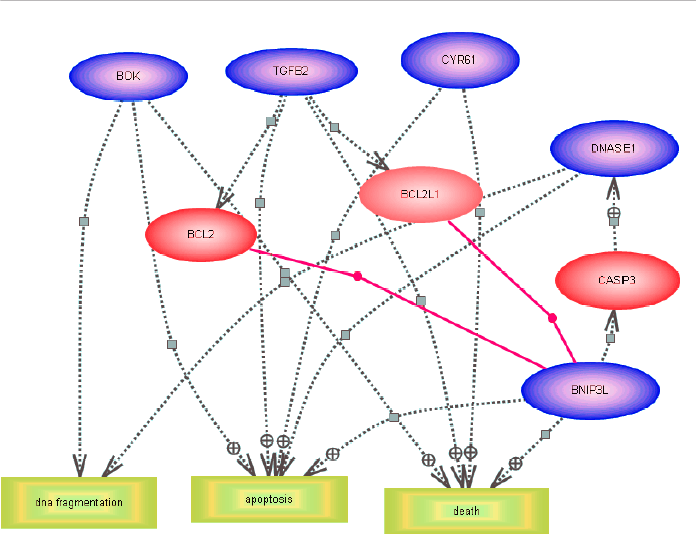
Biological pathway for the differentially expressed neuronal cell death genes in serum-deprived retinal ganglion cells. Pathways were identified by incorporating the microarray results (genes which are differentially expressed at 24, 48, and 96 h) into the Pathway Assit software. The pathway was constructed on this software by searching for the shortest path to connect the genes of interest by other genes or cell processes with which they interacted through expression or regulation only. Three major biologic processes are identified (apoptosis, death, and DNA fragmentation) and are represented by yellow rectangles. Blue ovals denote genes identified as neuronal cell death, and red ovals new genes connected to this pathway.

Five differentially expressed genes were also imported into Pathway Assit software (Figure 5). These had been previously reported to be specifically associated with neuronal cell death and to have altered expressions following serum deprivation for 24, 48, and 96 h. All five differentially expressed genes seemed to be associated with cell death. The genes involved in DNA fragmentation were *Bok* and *DNase 1* in addition to their association with death. The results indicated that these neuronal cell death genes were associated with three cell processes that included DNA fragmentation, death, and apoptosis. All these neuronal cell death genes were associated with apoptosis and they were also related to other target genes in the cell death pathway. There are three candidate genes reported to be connected to this pathway, which are *Bcl2, Bcl2l*, and *casp3*. We suggest that the limited number of genes involved in neuronal cell death that showed expression changes after serum deprivation may have been due to the limited numbers of annotated genes in the rat genome and the restricted conditions for data analysis. A stringent p-value had been used in these analyses.

### Annotation

To better understand the biological significance of these data, 39 differentially expressed genes at the 24, 48 and 96 h time points were annotated with the aid of software from the Gene Ontology Consortium (eGO Version 1.0). The data presented here (Appendix 1) show the GO annotations for the 39 differentially expressed RefSeq genes were common to the last three time points. The GO tool [39] has annotated genes in several model organisms that comprise three related hierarchies, including molecular functions, biological processes and cellular locations. For example, six of the 39 genes are located on the same chromosome (7q): [H1 histone family, member (NM_012578); Aquaporin 5 (NM_012779); calcium channel, voltage-dependent, beta 3 subunit (NM_012828); high mobility group AT-hook 2 (NM_032070); lectin, galactoside-binding, soluble 2 (NM_133599); and NADH-cytochrome b5 reductase (NM_138877)]. However the subcellular localization and functions of the gene products are quite different (Appendix 1). Mapping genes to the GO database is a useful tool for the automated extraction of useful biological information from gene expression data.

### Validation of microarray data

**Figure 6:**
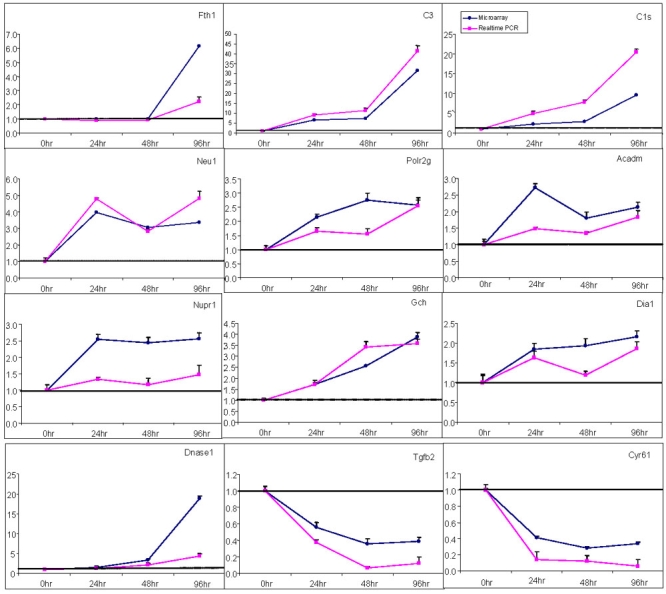
Comparative evaluation of microarray and real-time RT-PCR results. Twelve genes, identified as differentially expressed in gene arrays and regulated in response to time-dependent serum deprivation, were evaluated with RT-PCR. Values on the y-axis represent the fold change derived from the mean expression value for each gene, and values on the x-axis represent the time course of RGC-5 serum deprivation. Total RNA isolated from these cells was used for both microarray analysis and real-time RT-PCR. The closed circles (rectangle) represent microarray results, whereas the closed squares (circle) represent the quantitative RT-PCR data.

Real-time RT-PCR assays were performed for 14 genes whose expression levels were either upregulated or downregulated at the 24, 48, and 96 h time points (Figure 6). The genes include *Ftl1, C3, C1s, Neu1, Polr2g, Acadm, Nupr1, Gch, Dia1, DNase1, Tgfb2, Cyr61, Cyp2a3a*, and *Lagls2*. The expression of all genes was normalized to the 18S ribosomal RNA (housekeeping gene). Our real-time analysis of the 18S gene expression revealed that it was unaffected by serum deprivation since the difference in C_T_ values between apoptotic and nonapoptotic samples were negligible, which justified the use of 18S for normalization. There was an absence of RT-PCR product in samples in which the reverse-transcription step was omitted, which indicated that the results obtained represented true amplification of mRNA molecules. Overall, the gene expression trends by real-time RT-PCR concurred with the microarray data for all genes except a few, although the magnitude of changes differed (the only discordant genes were *Cyp2a3a, Lagls2*). The differences in relative expression by these two techniques may be attributed to their differences in kinetics and sensitivity. In general, relative expression changes by microarray analysis were greater than those by real-time RT-PCR, especially for genes that were markedly over-expressed. This may be explained by the better sensitivity, and perhaps, the better reliability, of microarray compared to the real-time RT-PCR method. RT-PCR was performed in parallel for genes differentially expressed at each time point to confirm or support the detected changes of expression levels. If the gene expression ratio is higher than 1, it is considered upregulated and if it is lower than 1, it is considered downregulated.

The data gathered from our microarray data and real time RT-PCR results indicated that C3 is significantly upregulated in RGC-5 serum deprivation. A recent study indicated that factor H inhibits the alternative pathway of complement activation, and a critical regulator of C3 activation in vivo, as well as factor H-deficient mice (*Cfh*^-/-^) mice spontaneously develop membranoproliferative glomerulonephritis (MPGN) that depends on C3 activation [40]. The data from this study showed that complement factor H (CFH) expression value were down-regulated about 0.6 fold in 48 h and 0.69 fold in 96 h during RGC-5 serum deprivation. Therefore, CFH was further investigated in RGC-5 and adult rat retina.

### Confocal microscopy

**Figure 7:**
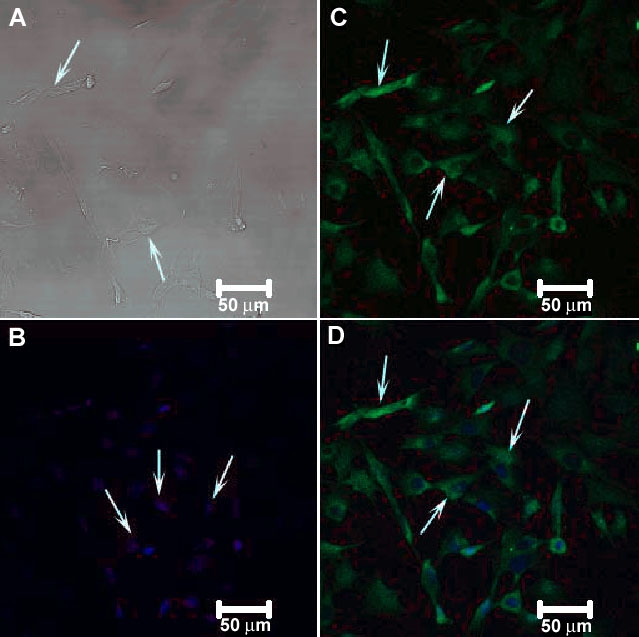
Confocal immunocytochemical localization of complement factor H (CFH) in RGC-5. The DIC image is shown in (**A**) and the nuclear staining by Hoechst stain is shown in (**B**). Arrows in **A** and **B** depict the cells and the nuclei of the RGC-5 cells, respectively. Complement factor H (CFH) in green (arrows in **C**). The merged images of these RGC-5 cells are shown in **D** (arrows). CFH was expressed in the cytoplasm (arrows in **B** and **D**).

**Figure 8:**
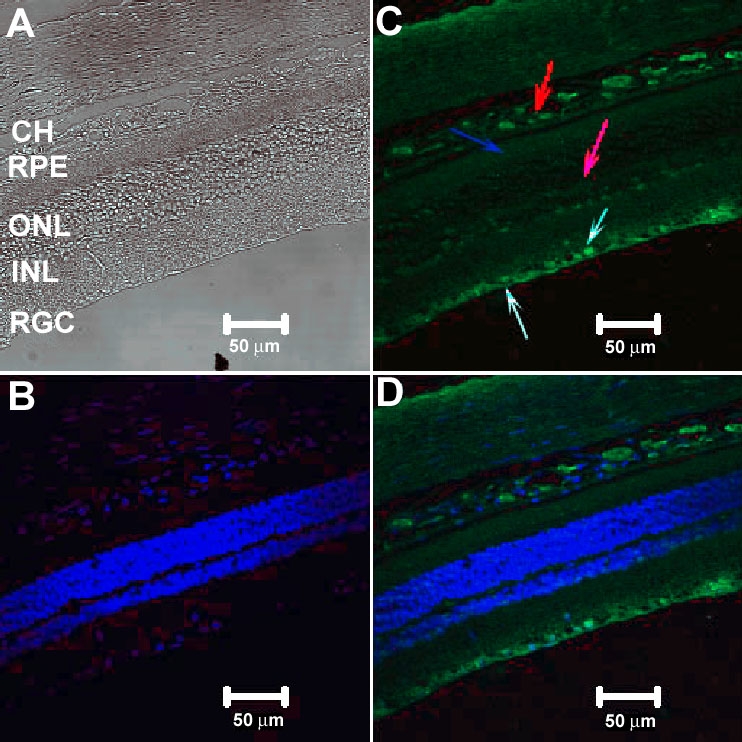
Confocal immunocytochemical localization of complement factor H complement factor H in the adult rat retina. A DIC image of retina showing various layers of rat retina (**A**). The nuclear staining by Hoechst stain is shown in (**B**). Intense factor H immunolabeling (**C**) was detected in retinal ganglion cell (RGC) layer (white arrow) including the nerve fiber layer (fluorescent blue arrow). Factor H labeling was also detected in the inner plexiform layer (purple arrow), photoreceptors (blue arrow), and the choroids (red arrow). The merged image of **B** and **C** is shown in **D**. INL indicates inner nuclear layer; ONL indicates outer nuclear layer; RPE indicates retinal pigment epithelium layer; and CH indicates choroids.

To examine the expression pattern of CFH in RGC-5 cells and rat retina, we used antibody to CFH. The RGC-5 cells (Figure 7C) showed intense factor H immunoreactivity in the cytosol with no reactivity in the nucleus. Merged images of factor H and Hoechst nuclear stain immunofluorescence clearly showed that factor H was expressed in the cytosol of the transformed RGC-5 cells (Figure 7D). As expected, there was no positive immunoreactivity with goat IgG controls (data not shown). For the adult rat retina, the immunofluorescence labeling of factor H in the retina (Figure 8) was observed predominantly in the RGC layer (white arrows in Figure 8C) and nerve fiber layer (fluorescent green arrow in Figure 8C) with positive immunolabeling in the photoreceptor cells (blue arrow in Figure 8C), RPE (light blue arrow in Figure 8C), choroids (red arrow in Figure 8C), and inner plexiform layer (purple arrow in Figure 8C). A diffuse labeling was observed throughout the thickness of the retina. The differential interference contrast (DIC) image of the retina is shown in Figure 8A. The retina was also stained with Hoechst nuclear stain (Figure 8B), and the merged image for factor H and Hoechst nuclear stain is shown in (Figure 8D). As expected, there was no positive immunoreactivity in IgG control retinas (data not shown).

## Discussion

Previous studies have suggested that serum deprivation induces apoptosis of RGC-5 cells through activation of mitochondrial signaling pathways [23]. The percentages of dying cells in serum-deprived media were shown here to be 9% at 8 h, 20% at 24 h, 48% at 48 h, and 85% at 96 h, and serum deprivation resulted in the upregulation of the pro-apoptotic Bax (NM_0.017059) and the downregulation of the anti-apoptotic BcL-2 (NM_016993). These data are consistent with our previously published [23] findings. The aforementioned studies were further extended here through use of gene microarrays, which allow for the simultaneous analysis of thousands of transcripts. This approach provides a larger view of the gene-regulatory networks involved in this particular model of retinal cell death.

Previously, most microarray studies have used a two-point system in which changes in expression between two different (control and experimental) states were compared, however, in this study, we used multiple time points. This approach led to the identification of some genes (Table 1) that were common to all time points, thereby enhancing their likely biological significance, and these are the primary focus of this report. There were many genes (Table 2) which were changed relative to a single time point. The presence of such changes may be indicative of the different phases of cell death as well as the activation of other cellular processes. These latter findings have not been extensively addressed in this report.

Ahmed et al. [32] recently reported that the complement component, C3, was upregulated after elevation of IOP in the rat eye by 8.5 fold at eight days and 10.5 fold at 35 days. Furthermore, several immune response genes were shown to be upregulated in monkeys with glaucomatous eyes [41]: C3 (1.9 fold), C3r (1.9 fold), and C1q (1.9 fold). Therefore, it was particularly noteworthy to see that the complement system was also activated in serum-deprived RGC-5 cells. A subset of differentially expressed genes identified in microarray data was validated using QRT-PCR (Figure 6). The present studies showed that the complement component, C3, is consistently up-regulated in serum-deprived cells by 6.5 fold at 24 h, 7.3 fold at 48 h, and 31.6 fold at 96 h. This dramatic increase in C3 was validated with RT-PCR (Figure 6). The complement components have been implicated in several brain disorders, especially where neurodegeneration is evident. For example, elevations in C3 mRNA expression have been demonstrated in patients with Alzheimer's disease [42-44]. Recent studies have suggested that the complement system either directly or indirectly contributed to cell death [45]. In addition to C3, the C1s (NM_138900) subcomponent of complement was consistently upregulated by 2.2 fold (24 h), 2.9 fold (48 h), and 9.6 fold (96 h) in serum-deprived RGC-5 cells. This subcomponent is responsible for cleavage of C4 and C2, which then triggers activation of the classical pathway [46]. The elevated levels of several complement components shown here indicate that RGC-5 cells are capable of synthesizing these components. Future studies should examine this possibility in the RGCs in animal models, and in the eyes of patients who have blinding diseases, because this would open a new door to the development of treatments for such diseases. While the retina, and the nervous system as a whole, has traditionally been seen as separate from the immune system, there has been increasing evidence over the last decade or two that glia mediate the immune functions of the nervous tissues. It now seems possible that neurons also have their own innate capacity for immune functions [47].

The present study indicates a decrease in the levels of survival promoting genes such as Akt. There were three members of the Akt gene family that were affected in the present studies: Akt1 (NM_033230); Akt2 (NM_017093); and Akt3 (NM_031575). They were down-regulated in RGC-5 cells after 96 h serum-deprivation by 0.69 fold, 0.55 fold, and 0.54 fold, respectively. The downregulation of Akt1, observed in all serum-deprived RGC-5 samples is consistent with results obtained by Miyahara et al [41] in which *Akt1* was downregulated in monkeys with mild and severe glaucomatous retinas. Akt may participate in constitutive survival processes in retinal neurons [48]. The Akt genes shown here, *Akt1* [49],  *Akt2* [50],  and *Akt3* [51],  are located on chromosomes 14q32, 19q13, and 1q44, respectively. All are widely, but differentially, expressed in various tissues. Members of the *Akt* family are important mediators of cell survival and have been shown to suppress the apoptotic death of a number of cell types induced by a variety of stimuli, including growth factor withdrawal [52-55]. Thus, Akt may promote cell survival through inhibition of a component of the cell death machinery [54].

Serum-glucocorticoid kinase (SGK) is downregulated at three time points (24, 48, and 96 h) of serum deprivation (Table 2). SGK, a 49-kDa serine/threonine kinase shares considerable structural similarity with Akt [56,57]. Akt-1 and SGK-1 are homologous kinases that are important downstream effectors of PI3-K signaling [58]. The PI3-K/Akt signaling plays a critical role in mediating survival signals in a wide range of neuronal cell types. These cells die through an apoptotic process after withdrawal of trophic factor [59]. In this regard, we do know that the use of blocking antibodies for BDNF and TrkB receptors leads to RGC-5 cell death [60].

The PI3-K pathway activation provides a strong protective effect through sustained activation of the downstream intermediary Akt, and the protective effect of Akt activation is mediated primarily through phosphorylation of BAD [61]. Our results showed that the expression of BAD, a pro-apoptotic member of the Bcl2 family increased 2.0 fold at 24 h and 2.2 fold at 96 h in serum-deprived conditions, a finding that is compatible with the decreased of Akt expression.

In this study, the upregulation of complement proteins and the downregulation of Akt suggested that these two systems may be interrelated. Rus et al. [62] reported that cell survival enhanced by C5b-9 is mediated by the PI3-kinase/Akt pathway, which inhibits apoptosis through regulation of BAD. Schematic signal transduction pathways involved in cell proliferation and survival induced by C5b-9 mediated through RAS, P13-K, Akt and BAD pathways has been previously reported [62].

It has been reported that CFH gene, an important regulator of the alternative complement cascade, is associated with age-related macular degeneration (AMD) [63]. Since Cfh is the main regulator of C3 activation [40]. Here we report that the Cfh (NM_130409) expression was significantly downregulated at 48 h 0.60 fold (p-value of 0.0077) and 96 h 0.69 fold (p-value 0.0279) in serum-deprived samples. Confocal microscopy studies reveal that complement factor H immunoreactivity was present in RGC-5 (in the cytosol), while in the rat retina, it was observed in the RGC layer and nerve fiber layer.

Neurons have the capability to express the mRNAs of complement proteins in vivo [42,47]. Complement proteins have been implicated in several brain disorders including neurodegenerative diseases [47],  such as in the brain with Creutzfeldt-Jakob disease [64],  and in Alzheimer's disease [43,44]. CFH, the major inhibitor of the alternative complement pathway, was a constituent of retinal lesions (drusen) in human donors with a history of AMD [63]. Complement protein plays an important role in immuno-regulation and may also exert pro- or anti-apoptotic effects in various cell types [65]. The complements system is receiving increasing attention as it becomes more important in the pathogenesis of tissue injury following immune, ischemic, or infectious insults [66]. Abnormalities, of Cfh have been implicated in MPGN in both human and experimental animal models [43]. Mice deficient in factor H (*Cfh*^-/-^ mice) developed MPGN spontaneously and were hypersensitive to developing renal injury caused by immune complexes [40].

In conclusion, we showed the use of immortalized RGC-5 cells to study a high-throughput genome-wide gene expression profile for serum-deprived cells. We propose that the patterns of expression reflect the withdrawal of trophic factors associated with the serum. This approach is more suitable to contribute to a realistic view of the complex series of events that terminate in cell death than looking solely at the effects of individual genes. We believe that the differentially expressed genes identified in both microarray and real-time RT-PCR techniques most likely play an important role in RGC-5 cell death due to serum deprivation. These data implicate a role for increased expression of complement components and a reduced expression of complement factor H and Akt in RGC-5 cell death.

## Appendix 1. GO data.

Thirty-nine differentially expressed genes at the 24, 48, and 96 h time points were annotated with the aid of software from the Gene Ontology Consortium (eGO Version 1.0). The GO annotations for the 39 differentially expressed RefSeq genes were common to the last three time points. The GO tool [39] has annotated genes in several model organisms that comprise three related hierarchies, including molecular functions, biological processes and cellular locations. These data are included in the file "Appendix 1". To access these data, click or select the words "GO data". This will initiate the download of a compressed (zip) archive. This file should be uncompressed with an appropriate program (the particular program will depend on your operating system). Once extracted, you will have a file that is tab delimited text. Most spreadsheet programs will import files in this format.
